# Room Temperature Ferromagnetic Mn:Ge(001)

**DOI:** 10.3390/ma7010106

**Published:** 2013-12-27

**Authors:** George Adrian Lungu, Laura Elena Stoflea, Liviu Cristian Tanase, Ioana Cristina Bucur, Nicoleta Răduţoiu, Florin Vasiliu, Ionel Mercioniu, Victor Kuncser, Cristian-Mihail Teodorescu

**Affiliations:** National Institute of Materials Physics, Atomistilor 105b, Magurele-Ilfov 077125, Romania; E-Mails: adrian.lungu@infim.ro (G.A.L.); laura.stoflea@infim.ro (L.E.S.); liviu.tanase@infim.ro (L.C.T.); cristina.bucur@infim.ro (I.C.B.); nicoleta.radutoiu@infim.ro (N.R.); fvasiliu@infim.ro (F.V.); imercioniu@infim.ro (I.M.); kuncser@infim.ro (V.K.)

**Keywords:** molecular beam epitaxy (MBE), Mn-Ge ferromagnetism, low energy electron diffraction (LEED), scanning tunneling microscopy (STM), high resolution transmission electron microscopy (HRTEM), X-ray photoelectron spectroscopy (XPS), magneto optical Kerr effect (MOKE), superconducting quantum interference device (SQUID) magnetometry, diluted magnetic semiconductors (DMS)

## Abstract

We report the synthesis of a room temperature ferromagnetic Mn-Ge system obtained by simple deposition of manganese on Ge(001), heated at relatively high temperature (starting with 250 °C). The samples were characterized by low energy electron diffraction (LEED), scanning tunneling microscopy (STM), high resolution transmission electron microscopy (HRTEM), X-ray photoelectron spectroscopy (XPS), superconducting quantum interference device (SQUID), and magneto-optical Kerr effect (MOKE). Samples deposited at relatively elevated temperature (350 °C) exhibited the formation of ~5–8 nm diameter Mn_5_Ge_3_ and Mn_11_Ge_8_ agglomerates by HRTEM, while XPS identified at least two Mn-containing phases: the agglomerates, together with a Ge-rich MnGe_~2.5_ phase, or manganese diluted into the Ge(001) crystal. LEED revealed the persistence of long range order after a relatively high amount of Mn (100 nm) deposited on the single crystal substrate. STM probed the existence of dimer rows on the surface, slightly elongated as compared with Ge–Ge dimers on Ge(001). The films exhibited a clear ferromagnetism at room temperature, opening the possibility of forming a magnetic phase behind a nearly ideally terminated Ge surface, which could find applications in integration of magnetic functionalities on semiconductor bases. SQUID probed the co-existence of a superparamagnetic phase, with one phase which may be attributed to a diluted magnetic semiconductor. The hypothesis that the room temperature ferromagnetic phase might be the one with manganese diluted into the Ge crystal is formulated and discussed.

## 1. Introduction

Diluted magnetic semiconductors (DMS) have been widely studied for more than two decades, and amongst these systems Mn*_x_*Ge_1−_*_x_* is a promising candidate owing to its high Curie temperature [1,2]. Surface science techniques often succeed in stabilizing metastable phases of manganese with ferromagnetic properties [3–5]. Solid state compounds such as Mn_13_Ge_4_, Mn_11_Ge_8_, Mn_5_Ge_2_, Mn_5_Ge_3_, Mn_3_Ge_2_ are known to date [6], of which only Mn_5_Ge_3_ has been reported to be ferromagnetic. This compound seems to be the origin of the detected ferromagnetism in Mn-Ge diluted magnetic semiconductors [7–9]. Hence, despite the relative success in stabilizing ferromagnetic Mn-based phases on Ge(111) [4,5,7,8,10], it is highly desirable to find a simpler route to synthesize almost macroscopic Mn-based compounds, possibly richer in germanium in order to provide less metallic character and to be more appropriate for integration with Si-based electronics.

Some recent reports demonstrated enhanced magnetic properties solely by thermal effects [11], therefore annealing to promote Mn interdiffusion with Ge could be a valid alternative to provide more DMS-like compounds. Even superparamagnetic Mn-Ge compounds [12] could be very appropriate for applications in magnetic sensors directly integrated on semiconductors [1]. Note also that an interesting surface for technology is Ge(001), whereas the majority of studies that have succeeded in stabilizing Mn-based ferromagnetic compounds so far proceeded with the Ge(111) surface.

The soundest recent reports of room temperature ferromagnetism on Ge(001) based systems occured via a “subsurfactant epitaxy” method subsequent to the deposition of half a monolayer of Mn on Ge(001) [5]; however, in this last case the relatively low Mn content obtained (about 0.25%) precludes further applications in magnetic devices. The synthesis of a Ge-rich phase (~MnGe_2_) was also achieved, organized in nanocolumns, providing a Curie temperature over 400 K [13]. A previous work on such columnar structures did not succeed in synthesizing room temperature ferromagnetic material [5,14], which points out the quite delicate conditions required in using co-evaporation of manganese and germanium on Ge(001) in order to achieve robust magnetism.

A simpler route for synthesizing such magnetic systems is the so-called “solid phase epitaxy” [9,15], where Mn is simply deposited on Ge single crystals either at room temperature, with subsequent annealing, or directly at a more elevated temperature. So far, results have been reported on Ge(111) surfaces [4,15–18]. A Mn_5_Ge_3_-induced superstructure of ( $\sqrt{3}\times \sqrt{3}$)*R* 30° was observed by reflection high energy electron diffraction (RHEED) in [4,15], and by low energy electron diffraction (LEED) in [16], whereas all references exhibit a strong decrease of the saturation magnetization near room temperature. Nevertheless, room temperature ferromagnetism was reported in references [9,16,18] and a weak magnetic moment at room temperature seems also to be provided by the studies reported in reference [15]. Comparatively, there are no reports on surface structure or eventual reconstructions, nor on magnetic properties for Ge(001) subject to solid phase epitaxy, despite the fact that this surface is similar and may be connected to the technologically important Si(001) surface. In this work, we report on LEED and scanning tunneling microscopy (STM) observations, together with magneto-optical Kerr effect (MOKE) and superconducting quantum interference device (SQUID) measurements on Ge(001) subject to Mn deposition at relatively high temperatures (250–350 °C). Below 250 °C substrate temperatures, no room temperature ferromagnetism is detected. The preservation of the Ge(001) surface upon deposition of a considerable amount of Mn (the equivalent of 100 nm of bulk manganese) is observed, together with a ferromagnetic hysteresis loop at room temperature.

X-ray techniques on MnGe systems have been applied extensively during recent years: (a) Mn L_2,3_-edges X-ray absorption (XAS) and X-ray magnetic circular dichroism (XMCD) for deriving magnetic moments [4,9,10,17,19] and (b) X-ray photoelectron spectroscopy (XPS) [4,5,8,9,17]. In our contribution, we present a detailed analysis by curve fitting of XPS data for Mn grown on Ge(001), in a quite similar way to that presented for Ge 3d in reference [17]. In addition to the Ge 3d level, the fitting of the Ge 2p and Mn 2p levels will be discussed. These analyses are directly correlated with the occurrence of room temperature ferromagnetism in the Mn-Ge(001) layers observed and with high resolution transmission electron microscopy (HRTEM) observations, which also point to the formation of nanoscopic Mn_5_Ge_3_ agglomerates in this case.

## 2. Results and Discussion

### 2.1. Low energy Electron Diffraction (LEED)

**Figure 1.**
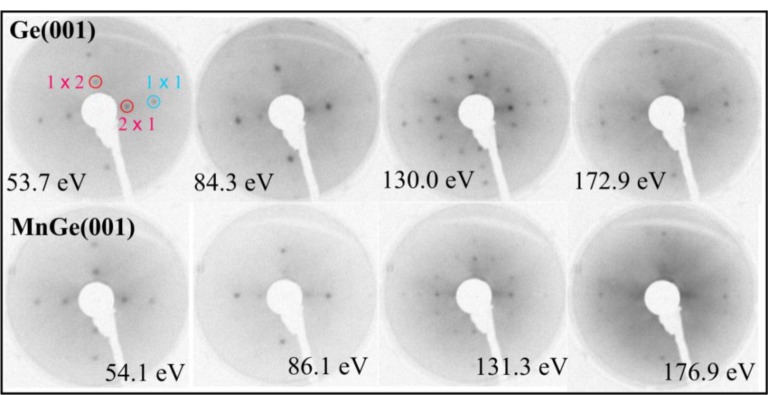
Low energy electron difraction (LEED) patterns for Ge(001) on the panels above, and for Mn deposited on Ge(001) at 350 °C (panels below). Each image is indexed with the energy of incoming electrons. For better clarity, negative images of the true LEED photographs are displayed. The (1 × 1), (2 × 1) and (1 × 2) spots are highlighted on one LEED image for clean Ge(001).

Figure 1 presents LEED patterns obtained on clean Ge(001) and after depositing the equivalent of a 100 nm thick bulk Mn layer on the substrate held at 350 °C. It is now clear that even the surface Ge(001) reconstruction subsists after Mn deposition. The LEED patterns obtained in the low energy regime are practically the same for Ge(001) and MnGe(001); only at higher electron kinetic energy (above ~ 150 eV) is the LEED pattern of MnGe(001) degraded with respect to the patterns obtained on clean Ge(001). Therefore, the first conclusion is that MnGe(001) samples present a high degree of crystallinity, quite similar to that of Ge(001). This is one of the few reports to our knowledge where a quite thick metal film is deposited onto a semiconductor and preserves its crystal structure and even its surface reconstruction. In the case of high temperature deposition of Fe on Si(001) (2 × 1)−(1 × 2) by using exactly the same setup, the LEED pattern vanishes starting with 1 nm of Fe thickness [20,21]; in the case of Sm/Si(001), a broad LEED pattern subsisted up to about 3.5 nm of Sm deposited [22,23]. A case closer to the present one was presented by Fe deposited in Ge(001) by keeping the substrate at high temperature (500 °C) [24], where the (1 × 1) LEED pattern of Ge(001) subsisted after 2 nm of Fe deposited; however, without providing also the (2 × 1)−(1 × 2) surface reconstruction of Ge(001). By taking into account all of the previous experiments, we can say that the observation of a Ge(001) surface quite similar to clean Ge(001), after a high amount of Mn deposited, is a new result. This suggests the strong migration of manganese atoms inside the Ge(001) single crystal.

### 2.2. Scanning Tunneling Microscopy

**Figure 2.**
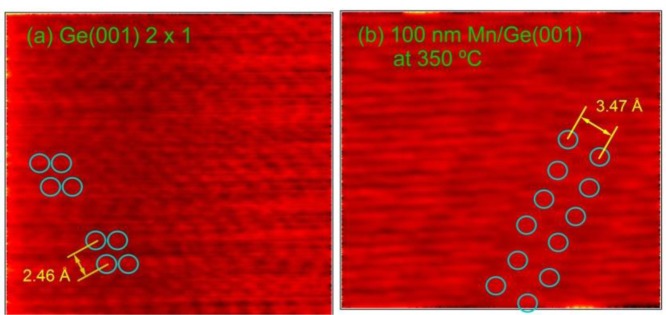
Scanning tunneling microscopy (STM) images obtained at a tip voltage of +150 mV (empty-states images), on (**a**) clean Ge(001) and (**b**) after the deposition of 100 nm Mn at 350 °C. The scanned area is 3 ×3 nm^2^ in both cases.

To gain more insight on the surface structure, STM images were taken on clean Ge(001) and on the sample obtained by a large amount (equivalent to a 100 nm film with bulk density) of Mn deposited at 350 °C. Figure 2 present two such scans. The Ge−Ge asymmetric dimers, separated by 2.46 Å [25] are visible on the clean Ge(001) surface [26,27]. Dimers are visible also on the surface obtained after Mn deposition-diffusion into the Ge(001) single crystal. Nevertheless, it seems that the interatomic distance in the dimers increases (it was measured as 3.47 Å) towards the Ge–Ge distance for an unreconstructed Ge(001) surface (about 4.0 Å). However, these dimer rows are sufficient to provide the (2 × 1)−(1 × 2) reconstruction of the Ge(001) surface, as detected by LEED. Note also that repeated STM scans proved a high degree of homogeneity and a relative flat surface after Mn deposition.

### 2.3. High-Resolution Transmission Electron Microscopy

**Figure 3.**
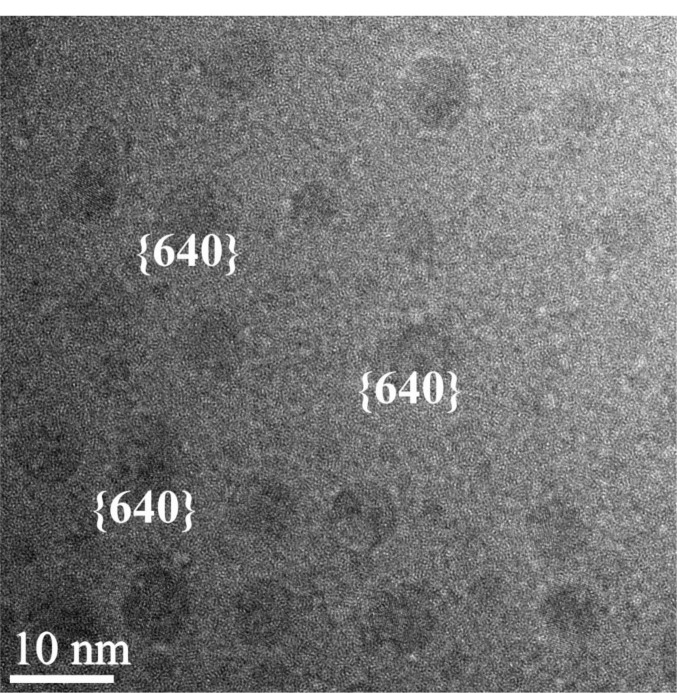
Cross section high resolution transmission electron microscopy (HRTEM) image of molecular beam epitaxy (MBE) MnGe layer deposited at 350 °C.

In a cross specimen, prepared for a substrate temperature of 350 °C, randomly distributed Mn_11_Ge_8_ spherical precipitates, as identified by selected area electron diffraction (SAED), with sizes ranging between 5 and 8 nm, are clearly visible (Figure 3).

**Figure 4.**
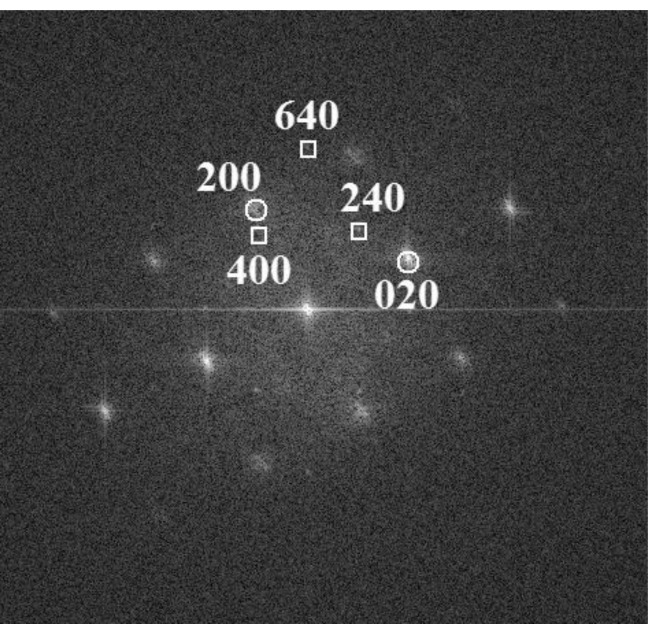
The associated fast Fourier transform (FFT) pattern to a plane view of MBE MnGe layer deposited at 350 °C (open circles—Ge matrix; square—Mn_11_Ge_8_).

The associated fast Fourier transform (FFT) of a plane view of the same specimen (Figure 4) reveals lattice spacings amounting to 0.345 nm, 0.327 nm and 0.191 nm, which correspond to Mn_11_Ge_8_ (240), (400) and (640), respectively. These values are in excellent agreement with the corresponding theoretical distances 3.40, 3.30, and 1.92 Å, respectively, for the orthorhombic Mn_11_Ge_8_ compound, with unit-cell parameters: a = 13.20 Å, b = 15.88 Å and c = 5.09 Å [28,29]. To further confirm the spherical precipitates belonging to the Mn_11_Ge_8_ phase, the crystallographic angles between the above mentioned atomic planes were measured and the results also fitted perfectly with the theoretical angles of this phase. Also, some clusters exhibit {640} planes identified by the interplanar distances of 0.191 nm (Figure 3). A zone axis pattern of (001) Ge is also present in the same FFT image shown by strong intensity spots (200) and (020). The crystallographic relationship between the Mn_11_Ge_8_ cluster and the Mn doped Ge matrix can be determined as (001)_Mn__11__Ge__8_||(001)_Ge_, which has not been reported until now. In a recent paper [30], no particular crystallographic orientation with respect to the Ge matrix was inferred for orthorhombic Mn_11_Ge_8_.

**Figure 5.**
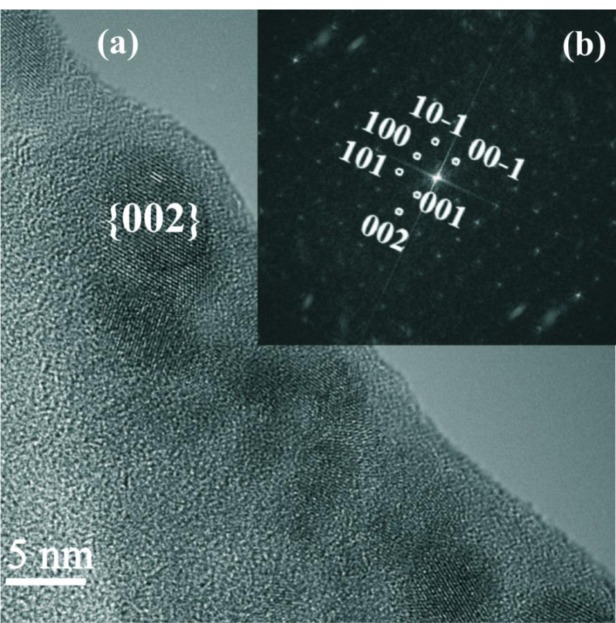
(**a**) Cross section HRTEM image of an area of MBE MnGe layer deposited at 350 °C and (**b**) the associated FFT pattern showing the presence of Mn_5_Ge_3_ (electron beam direction [10]).

In another cross section HRTEM image (Figure 5a), one can observe precipitates between 5 and 10 nm with a dark contrast and a Mn-rich phase formed near the surface during the film growth when a threshold Mn concentration was reached [31]. Some precipitates have (202) lattice planes parallel or normal to the macroscopic surface. Energy dispersive X-ray (EDX) analysis yields a Mn content around 5% whereas the Mn content in the matrix was found to be less than the detection limit of 1%.

Using the FFT image (Figure 5b) of the cluster crystal phase, shown in Figure 5a, we can conclude that it represents a reciprocal lattice section for HCP Mn_5_Ge_3_ in the [10] beam direction. The interplanar spacings of the reciprocal lattice points indexed in Figure 4b are 6.20, 5.08 and 3.90 Å, for the planes 100, 001, and 101, respectively. These values are in a very good agreement with the corresponding theoretical distances 6.22, 5.05 and 3.92 Å, respectively, for the Mn_5_Ge_3_ compound, with unit-cell parameters: a = b = 7.18 Å and c = 5.05 Å [32].

It is well known that the most stable phases in Mn-Ge alloys are ferromagnetic metals: Mn_11_Ge_8_ (T_C_ ≈ 270 K) and Mn_5_Ge_5_ (T_C_ ≈ 296 K). We must mention that some authors have found in MnGe layers prepared by MBE or in ion implanted MnGe films only the Mn_5_Ge_3_ compound [12,33–37] but there are a few papers which have identified both of the Mn_11_Ge_8_ and Mn_5_Ge_3_ crystal phases [29,30,38–40].

### 2.4. X-ray Photoelectron Spectroscopy

**Figure 6.**
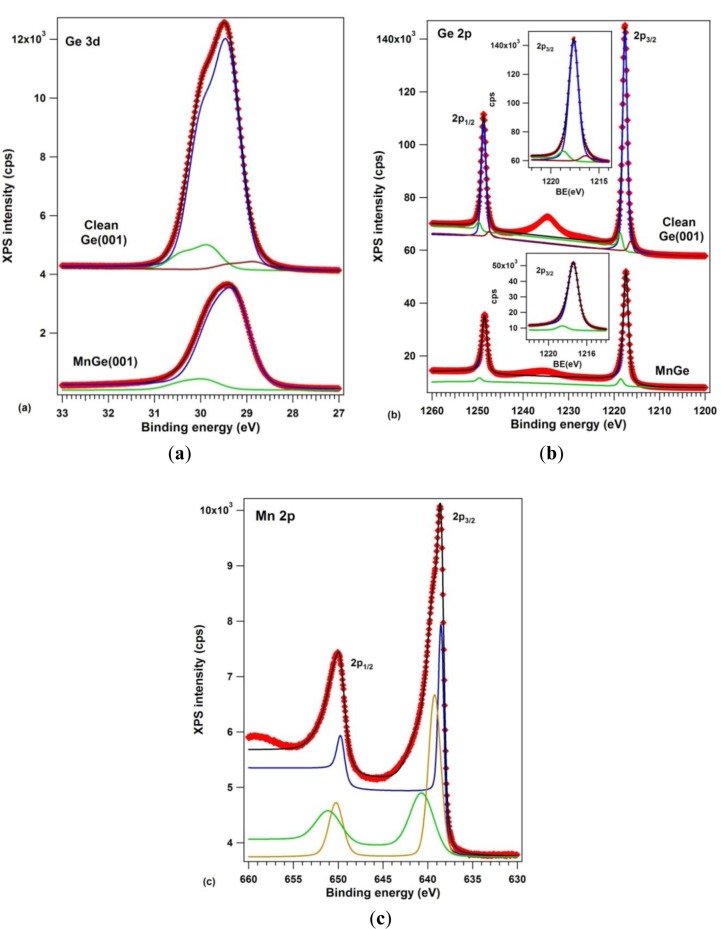
X-ray photoelectron spectroscopy (XPS) results: (**a**) Ge 3d core levels; (**b**) Ge 2p core levels and (**c**) Mn 2p core levels. For (**a**) and (**b**), the spectra obtained on a clean Ge(001) (2 × 1) are also represented. All spectra are fitted by using Voigt doublets and integral inelastic backgrounds (see the Experimental section for details). Inserts in (**b**) are detailed regions of the Ge 2p_3/2_ core level.

The samples were analyzed by X-ray photoelectron spectroscopy using one of the best resolutions achievable outside synchrotron radiation centers (well below 1 eV full width at half maximum certified on standard samples, see the Experimental section). Figure 6 presents the XPS data: (a) Ge 3d; (b) Ge 2p and (c) Mn 2p. Ge 3p and Mn 3p core levels were also measured, but discussion of them is not undertaken here. The clean Ge(001) core levels were simulated with three components, one for the bulk Ge, one for subsurface Ge atoms and one for Ge dimers [17,41]. This is the first time that both Ge 3d and Ge 2p spectra have been used in a compositional analysis of Mn-Ge systems. For Ge 3d, the spin-orbit splitting was found to be 0.585 ± 0.001 eV, practically the same as in references [17,42], and the branching ratio deviates slightly from its statistical value of 1.5, being 1.595 ± 0.005. Such deviations may arise from a wide variety of factors, including photoelectron diffraction effects [43]. For Ge 2p, the spin-orbit splitting obtained is 31.046 ± 0.003 eV and the branching ratio is quite close to its statistical value of 2. The integral amplitudes and binding energies are summarized in Table 1.

According to high resolution photoelectron spectroscopy data [41], for clean, (2 × 1)−(1 × 2) reconstructed Ge(001), the central component (1) at 29.413 eV is the bulk component, the component (3) at lower binding energy (28.820 eV) corresponds to the upper atom of the buckled dimer, which is slightly negatively charged because it has a dangling bond, whereas component (2), of higher binding energy (29.829) is attributed to the lower atom of the buckled dimer and eventually also to atoms from the subsurface layer. The data interpretation is similar to that of reference [17]. The same attribution is inferred also for the three components of the Ge 2p spectrum. The intensity ratios between components (2) and (3) to the bulk component (1) is roughly the same; namely the ratio between component (3) and the bulk component (1) goes from 3.8% to 5.4% from Ge 3d to Ge 2p, which is understandable, since Ge 2p electrons have lower kinetic energy (~270 eV compared to ~1460 eV) and are more surface sensitive [44]. The inelastic mean free path (IMFP) for Ge 2p is around 7–9 Å, and for Ge 3d it is about 16–18 Å [45,46].

By taking together the data for clean Ge(001), it seems that more reliable results might be obtained from Ge 3d than from Ge 2p when investigating the bulk properties, and by using Ge 2p when dealing with surface properties. Sometimes, one has to take into account also the fact that chemical shifts are larger for the Ge 2p core level than for Ge 3d. The only drawback in using Ge 2p levels is that, when working with Al K_α__1_ monochromatic excitation, one has to ensure that the sample is not at all contaminated with carbon, otherwise the C KLL Auger peak will be embedded in the Ge 2p structures. We mentioned in the Experimental part that special care was undertaken to work only with samples without measurable carbon or oxygen contamination. Also, despite the fact that the O 1s is embedded in the Ge LMM Auger electron mainfold, we carefully monitored the O 2s line, which is quite close to the Ge 3d line. The clean Ge(001) and all layers with manganese deposited on it did not show any oxygen contaminants within the XPS detection limit of 0.01% of a single atomic layer. Also, despite sometimes very long XPS acquisitions (exceeding one day of measurements), the Ge(001) sample did not get contaminated, a result which is in line with the quite low contamination rate induced by XPS on Si(001) [46,47], as compared with electron-based techniques (e.g., Auger electron spectroscopy).

When manganese is embedded in germanium, only two components are necessary to simulate both Ge 3d and Ge 2p spectra. A bulk Ge 3d component is identified at 29.243 eV, slightly shifted with respect to the case of clean Ge, which could eventually be attributed to a band bending effect [45]. This band bending is of about 0.17 eV towards higher values on an absolute scale, or lower binding energies. Following the assumptions illustrated in Figure 1 of reference [45] for typical cases of band bendings, see Figure 1 of reference [48] for explanation of the band bending assessment by using core levels, and Figure 1b,c of reference [49] for proof of these assessments, the band bending derived in the actual case deviates from the expected value due to the difference of workfunctions between Mn (4.1 eV) and Ge (5.0 eV) [50], which should induce a band bending towards higher binding energies, by some 0.9 eV. This confirms that no bulk metal manganese is formed on Ge(001), but rather a compound whose workfunction (~5.17 eV) is close to that of germanium. Additional proof is as follows: when Au is deposited on a similar Ge(001) wafer as used in the actual experiments at relatively low temperature, a band bending of the Ge 3d “bulk” component is obtained by ~0.15 eV towards lower binding energies [26], as expected from the difference of workfunctions between Au (5.1–5.3 eV) and Ge (5.0 eV) [50]. When Au is deposited at elevated temperature (750 K), the band bending reverses its sign: it yields about 0.1 eV towards higher binding energies [27], close to the actual case. The origin of this reversed band bending (irrespective of the workfunction of the metal used) suggests the presence of defects introduced by the metal under the Ge(001) surface, which act as shallow acceptors [51]. The main conclusion of all these considerations is that no metal manganese is formed on the Ge(001) surface, but only defects are induced in the subsurface layers of germanium.

A surface component in the Ge 3d spectra, upon Mn deposition, appears at an energy similar to component (2) of clean Ge(001). The lower binding energy component of clean Ge(001) is absent, indicating that there is no dangling bond on this surface. Also, the ratio between component (2) and the bulk component (1) is quite similar between clean Ge(001) and the case of Mn embedded in Ge. Similar findings may be inferred also from the Ge 2p spectra. Therefore, although the LEED pattern is similar, it may happen that the orientation of the dimers and their charge state is modified in the case of MnGe(001) with respect to the case of clean Ge(001), as was evidenced also for Au/Ge(001) [26,27]. This result is in line with the STM observations from Figure 2b, namely the different interatomic distance obtained for the surface dimers. A similar finding (absence of the lowest binding energy component) was reported for Au-induced chains on Ge(001) [41]. Note also that the fact that component (2) is a surface component may be derived also from the very low inelastic background associated with this component, as resulted from the fit [52,53] and as commented also in the Experimental Section.

The actual results are at variance with a recent published work on 16 ML Mn deposited on Ge(111) *c*(2 × 8) and annealed at 260 °C [54,55], where these layers exhibited surface components shifted towards lower binding energies by ~0.6 eV and ~0.4 eV. However, as the detailed LEED and STM data from the above reference proved, the surface in that case is a pure Mn germanide one, of $\sqrt{3}\times \sqrt{3}$ modulated superstructure, whereas in the actual case not only the initial surface is different, but also there is clear evidence of strong diffusion of manganese inside the Ge(001) wafer. Therefore, in the actual case the Ge surface component for the Mn-Ge film in Figure 6a,b might be attributed to the surface Ge layer, eventually to the ‘relaxed’ dimers evidenced by STM. Another interpretation is commented on a few paragraphs below, by associating this Ge component to the highest binding energy component of Mn 2p and attributing it to Mn_5_Ge_3_ clusters.

The Mn 2p XPS spectra may be simulated reasonably only with three components, as was proposed also in reference [55]; additionally, we needed to allow strong deviations of the spin-orbit splitting from its statistical value of 2, namely 2.85 for component (1), 2.34 for component (2) and 1.66 for component (3). We must again attribute this effect to photoelectron diffraction phenomena [43,56]. Also, most of the inelastic background is associated to component (1), of lowest binding energy (638.50 eV). According to actual data interpretations of these separate inelastic backgrounds [52,53], this indicates the bulk nature of component (1), the clear surface nature of component (2) and the eventual subsurface nature of component (3). Component (1) has a binding energy quite close to that of metal Mn [57], component (2) has a binding energy close to that of MnP (639 eV, according to [58]), whereas component (3) has an energy close to that of oxidized Mn (640 eV, according to [59]). We must emphasize that special care was undertaken to work on samples without any carbon or oxygen contamination. Although the O 1s peak is embedded in the LMM Auger manifold of Ge when working with Al K_α_, the O KLL and O 2s peaks are found in an energy region free for any other peak and no structure was observed in this region for all samples analyzed and presented in this work. Therefore, it is unlikely that component (3) can be attributed to any oxidized manganese.

In some older experiments, metal Mn was deposited at low temperatures on Ge(001) and resulted in a thick Mn film on germanium. Several hours of XPS analyses in UHV (4−5 × 10^−10^ mbar) induced an observable modification of the Mn 2p spectrum, with decrease of the main (metal) peak at 638.5–638.7 eV and appearance of the oxide peak at 640.0–640.5 eV. This was not the case in the actual experiment. The surface was quite stable after several hours of XPS measurements. This suggests that most manganese in the metal state is found in the form of metal Mn clusters or metal nanoparticles embedded deep inside the Ge(001) crystal, giving a component of lower binding energy, (1). HRTEM did not observe such nanoparticles with a crystal structure of manganese, therefore these metal nanoparticles should have a quite reduced dimension. Being inside the Ge crystal, these metal atoms or nanoparticles will not be subject to oxidation by the residual gas from the UHV chamber.

For computing the concentrations, we used Wagner’s atomic sensitivity factors (ASF) [60] (see Table 1). Previous quantitative analyses when using these factors have given reasonable results, applied to our setup [45,48,49,61]. Only for rare earths had these ASF to be slightly adjusted [22,23]. It may also be observed that the results obtained by using Ge 2p and Ge 3d do not deviate that much. The relative deviation of about 21% for clean Ge(001) may indeed be attributed to some systematic errors from the atomic sensitivity factors. However, the deviation is much lower for the Mn-Ge(001) sample; at the same time, it may be seen that the summed corrected intensities for Mn 2p and Ge 2p are quite close (within 8.3%) to the corrected intensity for Ge alone (Ge 2p) in the case of the clean sample.

A deconvolution procedure of the Mn 2p spectra similar to that shown in Figure 6c was presented in reference [55] where component (3), of highest binding energy, is attributed to a satellite whose origin is briefly commented as “due to fine features from various Mn atomic configurations”, and reference [62] is cited to support this assertion. Concerning, the computations for Mn 2p XPS spectra presented in reference [62], Figure 3a has a wrong energy scale, particularly the 2p_1/2_ level has a lower binding energy than the 2p_3/2_ level. Therefore, we cannot rely on such computations for attributing the present data. Without discarding the possible atomic origin (probably a different occupancy of the 3d orbitals) giving rise to the highest binding energy Mn 2p_3/2_ component, we present in the following a more “traditional” viewpoint, based on the comparison between Mn 2p and Ge 3d (or 2p) components.

The ratio between the Mn 2p (3) component and the Ge 3d (2) component is 1.75, whereas the ratio between Mn 2p (3) and the Ge 2p (2) is 2.75. If this Mn 2p (3) component is attributed to the observed Mn_5_Ge_3_ and Mn_11_Ge_8_ clusters by HRTEM, the ratio should range between 1.38 and 1.67. The IMFP for Mn 2p is around 12–14 Å [44]. The strong deviation in the Mn:Ge ratio with respect to the expected value obtained when using the Ge 2p level may be connected to the quite low IMFP (7–9 Å) of Ge 2p photoelectrons. Therefore, the signal originating from Ge atoms from an embedded cluster is strongly attenuated with respect to the signal originating from Mn atoms. From Figure 4 it may be seen that the clusters are located at ~1–2 nm below the Ge(001) surface. This implies the following relationship between the “observed” [Mn:Ge]_obs._ atomic ratio and the “real” ratio [Mn:Ge]_0_:

$${\left[\text{Mn}:\text{Ge}\right]}_{\text{obs}.}={\left[\text{Mn}:\text{Ge}\right]}_{0}\text{ exp}\left(\frac{d}{\lambda_{\text{Ge}}}-\frac{d}{\lambda_{\text{Mn}}}\right)$$ (1)

*d* being the distance from the Ge(001) crystal surface to the surface of the embedded clusters and λ*_x_* being the inelastic mean free path of photoelectrons emitted by the element X. Without entering into too much detail, a reasonable fit with formula (1) of both observed integral amplitude ratios (*i.e.*, by considering Mn 2p and either Ge 3d or Ge 2p) may be obtained with λ_Ge 3d_ ≈ 18 Å, λ_Ge 2p_ ≈ 9 Å, λ_Mn 2p_ ≈ 9 Å, *d* ≈ 8 Å and [Mn:Ge]_0_ ≈ 2. Therefore, an approximate stoichiometry of Mn_2_Ge can be inferred for the clusters. In fact, the ratio between the atomic concentration of manganese and germanium may be influenced, e.g., by the presence of Mn atoms in the outermost layers of the clusters. We conclude that, with a good approximation, these clusters may have the composition Mn_5_Ge_3_ (eventually also Mn_11_Ge_8_), as derived from HRTEM observations.

The remaining question is the attribution of components (1) and (2) of the manganese spectrum. As mentioned above, component (1) may be attributed to some interstitial manganese atoms or very small clusters, not forming bonds with germanium. Component (2), whose binding energy is very close, could also be attributed to a metal-like state of manganese. Eventually, components (1) and (2) may represent the same component, but which could be rather simulated by a Doniach-Sunjic asymmetric lineshape [63]. However, the main problem of this lineshape is that its integral is not defined (it diverges [44]), therefore it is difficult to obtain reliable integral amplitude components for this component alone. Therefore, irrespective of the nature of lineshape used, the main conclusion is the same, namely that these (1) and (2) components represent small metal manganese atoms or particles embedded in germanium. These atoms or small particles were not visible by HRTEM: their existence is proposed only by the XPS data. The ratio between Mn(1 + 2) and Ge(1) ranges between 2.48 (when using the Ge 3d levels) and 2.64 (when using the Ge 2p levels). Therefore, the Mn diluted into germanium near the surface is present in a relatively high amount, yielding a compound such as MnGe_~2.5_. This result is at variance with the observed concentration of manganese inside germanium in areas free of Mn_5_Ge_3_ and Mn_11_Ge_8_ clusters, by EDX analysis. Two hypotheses may be formulated to explain this discrepancy: (1) the XPS analysis is sensitive to a layer of about three times the IMFP, around 5 nm, whereas the EDX analysis was performed on areas situated at larger depths; (2) the EDX analysis commented on in Section 2.2 was performed on specific regions of the sample where Mn_5_Ge_3_ and Mn_11_Ge_8_ aggregates were clearly visible; this does not exclude that in some other areas of the sample a larger manganese composition may be found.

Note also that the overall intensity between germanium and manganese is close to 2. Recently, Ge_2_Mn-like phases were computed by first principle simulations [64]. The above reference proposes three possible sites for Mn insertion in such a structure: substitutional in Ge diamond lattice (S), tetrahedral interstitial in the Ge diamond lattice (T), and the α-phase of CsCl-like structure, similar to FeSi_2_. Without the HRTEM observations, it would be tempting from the XPS data to propose a mixture of these three states of manganese in a compound which has the overall composition Mn:Ge ≈ 1:2.

Finally, we must specify that the actual interpretation does not completely discard the hypothesis that the Mn(3) component is a purely atomic effect, as presented in reference [62]. In that case, the Ge(2) component would represent only the Ge dimers on the surface, as observed by LEED and STM. The conclusion would be that the resolution of the actual data does not allow one to discriminate between Ge in Mn_5_Ge_3_ clusters and Ge from the Ge(001) crystal. The Ge(1) component would represent both types of Ge atoms from the bulk of the sample. The reason why we favor the hypothesis of Ge(2) + Mn(3) forming MnGe clusters is that, if one considers that the whole Mn signal (6.85 eV × kcps) originates from these clusters, it will be associated with the whole Ge bulk signal (some 11.4 eV × kcps), therefore no signal is left for germanium from the Ge(001) crystal, outside the clusters. At the same time, such areas are clearly observed by HRTEM and some Ge photoemission intensity could belong to these regions.

We end the XPS section by concluding with the formation of Mn_5_Ge_3_-like clusters, superposed with distinct (spare) manganese in the metal state, diffused in the Ge(001) crystal. Similar findings were reported also in References [9,10,65–67], for preparation conditions different from the one used in the actual study, ranging from co-evaporation to ion implantation. Additionally, here we may estimate that the overall amount of unbound or diluted manganese in the near surface region, as investigated by XPS, is about twice as much in the diluted form with respect to the manganese found in Mn_5_Ge_3_ clusters.

### 2.5. Magneto-Optical Kerr Effect

**Figure 7.**
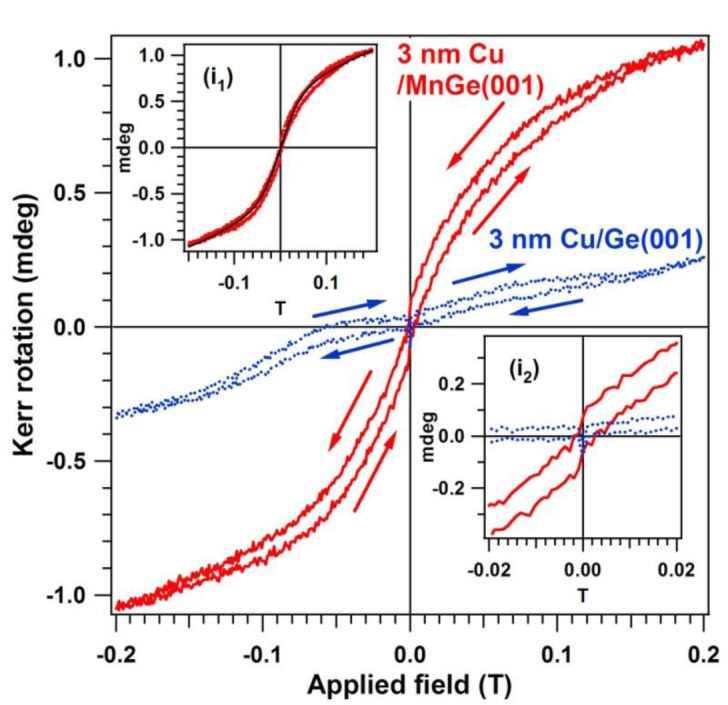
Magneto-optical Kerr effect (MOKE) hysteresis loops obtained on the sample with the equivalent of 100 nm Mn embedded in Ge(001), in red (full) curve, together with a measurement on a clean Ge(001) capped with the same amount of Cu as the Mn-Ge sample, in blue (dashed). Arrows represent the sense of evolution of the hysteresis (counter-clockwise for MnGe, clockwise for Ge). Insert (i_1_) represents a fit with a combinantion of Brillouin functions for assessing the superparamagnetic component. Insert (i_2_) represents a detail of the hysteresis loops in the low field region.

Figure 7 presents the MOKE hysteresis loop obtained on the MnGe(001) samples capped by a 2–3 nm Cu layer. In order to remove all possible effects that may give questionable results, a separate Ge(001) was prepared by flashing in UHV, checked by LEED and XPS, then covered by the same amount of Cu as the MnGe(001) samples, checked again (no LEED observed, no contamination seen by XPS). This was also investigated by MOKE and provided a loop with no ferromagnetic signal. We will not comment on the possible origins of the reverse (clockwise) hysteresis loop with a small inner area shown by this sample (blue curve in Figure 7).

A clear, counter-clockwise hysteresis loop is obtained for MnGe(001). This loop may be interpreted as the superposition of two paramagnetic components and one ferromagnetic component. A similar procedure to identify diamagnetic or paramagnetic components was discussed in reference [68] for cobalt doped ZnO. Simulations using the Brillouin function, described in more detail in references [20,21,69], pointed to the existence of a clear superparamagnetic component with total moment *J*_1_ ≈ 12,000 Bohr magneton (μ_B_) units, superposed on a “normal” paramagnetic component with *J*_2_ ≈ 5 μ_B_. However, in addition to these two components, a ferromagnetic behavior is clearly identified.

It is clear now that, according to the XPS observations, each magnetic component should be attributed to one of the Mn states identified. The most straightforward attribution is to assume that the observed Mn_5_Ge_3_ and Mn_11_Ge_8_ clusters are responsible for the ferromagnetic component, whereas the “diluted” Mn into the Ge matrix, whose binding energy is close to that of the metal manganese, should be responsible for the superparamagnetic component. An argument to propose this attribution is also that the ratio between the saturation magnetization of both components is about 10.6, in favor of the superparamagnetic component. From the XPS data analysis we inferred a ratio of about two between the metallike Mn and Mn from Mn_5_Ge_3_ clusters. If, in addition to that, one takes into account that the Mn atomic magnetic moment may reach 5 μ_B_ inside the superparamagnetic particles (according to Hund’s rules), whereas in Mn_5_Ge_3_ the ferromagnetic Mn moment is of about 2.6 μ_B_ [10], this could explain a ratio between saturation magnetization of about four. The remaining part until the observed value of 10.6 may be attributed to the fact that the compositions derived by XPS are valid in a narrow region of a few IMFP near the surface, *i.e.*, at most 2–3 nm, whereas the MOKE technique is sensitive to about 20 nm [70]. The main problem with this attribution is that the superparamagnetic Mn clusters should contain about 2400 atoms, *i.e.*, their volume (by introducing parameters for bulk manganese) should be about 29.3 nm^3^. Such clusters should have been visible by HRTEM.

The second hypothesis is more interesting for practical applications: to attribute the superparamagnetic component to the Mn_5_Ge_3_ clusters, and the ferromagnetic component to atoms or possibly small Mn clusters diluted into the Ge(001) crystal, diluted such that they cannot form distinct structures visible by HRTEM. One argument in favor of this hypothesis is that, when computing the cluster size starting with the total superparamagnetic momentum and by introducing the above value of 2.6 μ_B_ for each Mn atom from the clusters, one obtains on average about 4600 Mn atoms in a cluster, therefore the clusters are formed by about 7400 atoms. By assuming as a first approximation that, on average, the Mn atomic volumes are similar to the Ge ones, the total volume of such clusters yields in the range of about 90 nm^3^, *i.e.*, spheres with radii of about 2.8 nm—and these values are quite similar to the observed clusters by HRTEM (see Figures 2 and 4). Superparamagnetism (with or without interaction) of Mn_5_Ge_3_ clusters at room temperature has already been discussed in references [12,16,69]. SQUID measurements, which are detailed in the following, showed also the presence of a superparamagnetic together with a ferromagnetic component. Therefore, this hypothesis points out a very interesting result: the ferromagnetic component is indeed due to Mn diluted in the Ge matrix, therefore a diluted magnetic semiconductor is formed. It is reminded that the surface composition of this DMS may be obtained roughly by dividing the amplitude components (1) and (2) of the Mn 2p spectrum by the “unreacted” component (1) of Ge 2p: a composition of about 27 at% is obtained for the Mn content in the layer. An approximate composition of about Mn_2_Ge_5_ is derived near the surface. It is astonishing that, despite this huge amount of manganese, the Ge(001) surface remained unchanged.

### 2.6. SQUID Magnetometry

**Figure 8.**
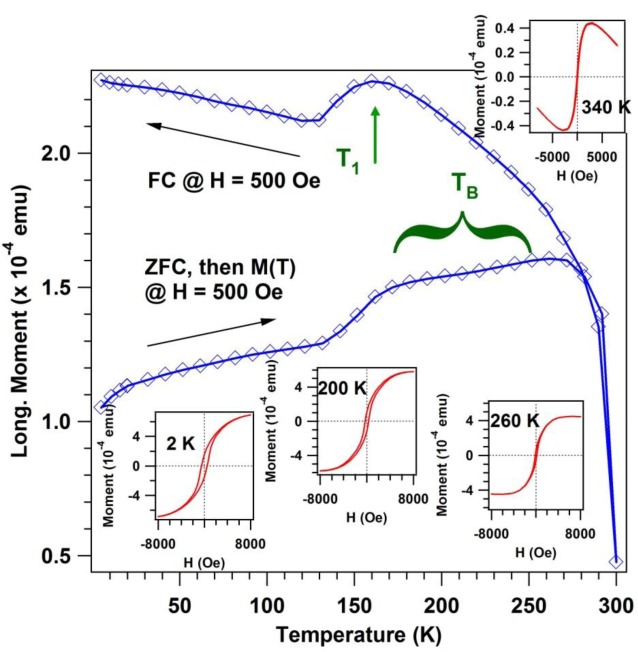
Superconducting quantum interference device (SQUID) measurement of the Mn-Ge(001) sample obtained by deposition of 100 nm Mn on Ge(001) held at 350 °C. The main graph with blue symbols and lines represent zero field cooled-field cooled magnetization measurements (see text for details). The inserts represent magnetization hysteresis measurements at the specified temperatures.

Figure 8 presents the SQUID data. Details about the measurements are given in the Experimental Section. First of all, let us remark that clear hysteresis loops are obtained at low temperatures (starting with 2 K) up to above room temperature, 340 K, although in this case the *M*(*H*) curves are dominated by the germanium diamagnetic component. From zero field cooled-field cooled (ZFC-FC) measurements the existence of a superparamagnetic component may be inferred [71] as a local maximum of the magnetization *versus* temperature dependence when the sample was first cooled without applied magnetic field and then heated in a small applied field. This maximum defines a “blocking temperature” above which the thermal energy suffices to overcome the magnetic anisotropy of the frozen macrospins. For a sample with a distribution of nanoparticle sizes, this blocking temperature manifests as a broad maximum or a plateau, as represented in Figure 8.

In addition to this superparamagnetic component, the ZFC-FC curves exhibit a clear presence of a ferromagnetic component, a result which is in line with the MOKE observations. Moreover, the FC curve exhibits also a maximum at a temperature *T*_1_ ≈ 160 K. Deviations from a ferromagnetic *M*(*T*) dependence are to be expected for diluted magnetic semiconductors [72] owing to the competition between thermal generation of carriers and thermally induced disorder in the spin alignment. Therefore, such an anomalous FC behavior may be a sign of a diluted magnetic character of the ferromagnetic component. The dependence of the coercive field with temperature (not shown) behaves as 1 − *T*/*T*_C_, a result reported already on similar samples in reference [73]. However, in this previous work *T*_C_ was in the range of 185–235 K, whereas in the actual case it is of about 335 ± 10 K.

We end up this section by estimating the average saturation magnetic moment per Mn atom from MOKE and SQUID measurements. According to previous calibrations of the MOKE setup, discussed in more detail in references [20–24,70], 1 mdeg of MOKE signal corresponds to 1 nm of a metal layer with typical density of bulk transition metals (around 10^23^ cm^−3^) and with 1 μ_B_ per atom. As mentioned above, the probing depth is of about 20 nm. With an average of 2 Mn atoms per Ge unit cell (5.66 Å)^3^, a density of 10^22^ Mn atoms per cm^3^ is obtained, *i.e.*, one order of magnitude lower than a typical density in a transition metal. By taking into account this density, an effective magnetic moment of about 0.5 μ_B_ per Mn atom is obtained at room temperature, normalized to all Mn atoms. Rescaling to retain only the components attributed to diluted manganese into Ge(001), yields about 0.75 μ_B_ per Mn atom diluted in germanium. Scaling again with the SQUID *M*(*T*) magnetization curve, a magnetic moment of about 3.3 μ_B_ is obtained at very low temperatures.

It may also happen that, of the Mn atoms, only a part of them are incorporated into the Ge(001) lattice and produce the diluted magnetic semiconductor (e.g., in substitutional sites (S), see reference [63]). Another part might quite well be responsible for the paramagnetic phase (e.g., in interstitial sites, (T)), which was also introduced in the Brillouin function simulation of the total magnetization.

## 3. Experimental Section

The experiments were performed in a surface science cluster (Specs) comprising a molecular beam epitaxy (MBE), an Aarhus scanning tunneling microscope and a photoelectron spectroscopy chamber. The system is described in greater detail in references [22,24]. The base pressure in all ultrahigh vacuum (UHV) chambers is in the low 10^−10^ mbar to 10^−11^ vacuum range. Photoelectron spectroscopies (XPS and spin- and angle-resolved ultraviolet photoelectron spectroscopy SARUPS) are performed in an analysis chamber equipped with a 150 mm Phoibos hemispherical electron energy analyzer, a dual anode (Mg/Al K_α_) X-ray gun, a monochromatized (Al K_α_/Ag L_α_) X-ray source and a high power UVS 300 UV lamp. A flood gun operating at 1 eV electron energy and 100 μA electron current was employed to ensure sample neutralization for all measurements. Monochromatized Al K_α__1_ radiation was used for this experiment (1486.74 eV). The analyzer operated in fixed analyzer transmission (FAT) mode with pass energy of 10 eV; the estimated combined (source + analyzer) resolution is of about 0.65 ± 0.03 eV. The energy was repeatedly calibrated with the Au 4f_7/2_ core level (83.81 eV) using separate thick Au depositions. The ARUPS and spin-resolved photoelectron spectroscopy is not discussed in this paper. STM measurements are performed at room temperature.

Ge(001) wafers were cleaned by flashing the samples at about 650 °C (2–3 flashes of 20 mins. in a vacuum kept in the low 10^−9^ mbar range) [24,26,27]. Clear (2 × 1)–(1 × 2) LEED were obtained, as seen in Figure 1. No carbon or oxygen contamination was detected within the limits of the XPS system (0.01% of a single atomic layer). Thick manganese layers (100 nm of equivalent bulk Mn) were deposited from a properly calibrated and outgassed Knudsen cell, close to normal incidence on the substrate, at a rate of 2 nm/min. Also, no contamination was observed. During the deposition, the substrates were held at various temperatures between 50 and 450 °C. Lower temperature deposition results in layers without any LEED pattern. Also, no room temperature ferromagnetism is observed for samples synthesized at temperatures below 250 °C. In this paper, we discussed mostly results obtained on substrates synthesized at 350 °C, where a high quality LEED pattern was obtained after the thick Mn layer deposition. The degree of Mn oxidation in the photoemission chamber was also estimated to be about one single atomic layer in 100 ± 15 minutes for a fresh Mn film. Several Mn 2p measurements as function of time elapsed in the analysis chamber were undertaken and no visible contamination of the manganese layer was observed.

Separate Mn/Ge(001) preparations were immediately covered by 1.5 nm of Cu and removed from the UHV system for *ex situ* analyses: X-ray absorption fine structure, SQUID, HRTEM, MOKE. Longitudinal MOKE was performed at room temperature by using a AMACC Anderberg and Modéer Accelerator AB system with a He-Ne laser (λ = 633 nm), which allows a maximum applied field of 0.6 T in the sample plane. In order to enhance the precision of the measurement in the low field region, a maximum field of 0.2 T was applied in these experiments, but samples were checked regularly also at maximum applied fields of 0.4 T.

Thermo-magnetic *M*(*T*) curves as well as magnetic hysteresis *M*(*H*) loops at different temperatures between 2 and 300 K were measured with a SQUID magnetometer (Quantum Design), with a magnetic field (up to 20 kOe for these samples) applied along the Ge(001) surface. The procedure to record ZFC curves is first to heat the sample at about 400 K, then to cool by not allowing it to experience any applied magnetic field down to the lowest temperatures achievable in the setup (2 K), then to apply a small magnetic field (500 Oe) and to record the magnetization *versus* temperature. For field cooled, the sample is cooled in applied magnetic field and the FC curve is measured during the cooling cycle; when it is warmed up, hysteresis cycles are recorded.

Other experiments undertaken were Cu 2p XPS measurements, deposited either on clean Ge(001) or on Ge(001) subject to Mn solid phase epitaxy: no difference was observed in these spectra, therefore no intermixing of Cu with the MnGe layers could be observed.

Cross-sectional HRTEM was performed by using a JEOL JEM ARM 200F microscope with Cs correction and 200 keV electron energy. The cross-sectional Cu-capped MnGe samples are mechanically thinned down to 20 μm by using the tripod method. Ion milling is achieved down to electron transparency by using a Gatan precision ion polishing machine (PIPS) machine, operated at 4 kV and 7° incidence angle. For the final milling stage, the acceleration voltage is reduced to 1 kV, in order to remove the damaged surface layer only.

The XPS data analysis was performed by simulations using Voigt lines and Voigt inelastic backgrounds [74]. As argued in references [52,53], associating separate background factors to each XPS component allows one to discriminate between the bulk and the surface nature of each one of these components. Also, the Ge 3d levels were fitted with the same Lorentzian and Gaussian widths for all lines, whereas for 2p spectra (Mn and Ge) the Lorentzian width was allowed to increase from the lower binding energy (2p_3/2_) to the higher binding energy (2p_1/2_) line, owing to supplementary Coster-Kronig decay channels, yielding lower core hole lifetimes for the higher binding energy lines [45,48,49,52,53].

## 4. Conclusions

A manganese based ferromagnetic system was stabilized on Ge(001), with preservation of the Ge(001) long range surface ordering. The synthesis method was a quite simple evaporation of Mn onto heated Ge(001) substrates, under controlled conditions of vacuum and temperature. The simultaneous analysis of LEED, HRTEM, XPS and MOKE data illustrated several characteristics of the MnGe(001) system:

(i) The Ge(001) surface crystal structure is preserved, together with the (1 × 2)−(2 × 1) reconstruction.
(ii) The atoms forming the surface dimers are situated at larger interatomic distances on the surface exposed to Mn.
(iii) Mn atoms are located in Mn_5_Ge_3_ and Mn_11_Ge_8_ clusters (about one third of them) and the other two thirds most probably diluted into the Ge(001) semiconductor lattice.
(iv) Three magnetic phases are present, one paramagnetic (5 μ_B_), one superparamagnetic (about 12,000 μ_B_) and one ferromagnetic. These states may be connected with the different Mn states observed in XPS. We presented some arguments, mainly based on HRTEM observations, that the superparamagnetic phase may be due to the observed clusters, whereas the ferromagnetic phase might be due to Mn diluted into germanium, unobserved by HRTEM.
(v) The sample surface is relatively insensitive to contamination by the residual gas, therefore most Mn atoms are located beneath the Ge(001) surface. This may be quite important for technological applications in microelectronics, where often the substrate is transferred from one processing system to another under ambient atmosphere.

The DMS character of the magnetic phase was considered, based on two observations: (a) the similitude between the “magnetic” size of the particles detected from the superparamagnetic component of the *M*(*H*) measurements; therefore, the ferromagnetic component might be due to manganese in other environments, e.g., diluted into the Ge(001) matrix, unobserved by HRTEM and (b) the maximum observed for the ferromagnetic component in the field cooled curve, which might be attributed to a DMS-like character. This hypothesis remains to be investigated in more detail, especially by complex magneto-electric or magneto-optical experiments, where the carrier concentration inside the Ge layer may be controlled. To check the truly inert nature of the Mn states, one has also to try controlled oxidations or other contaminations of these surfaces. However, the actual data, apart from boosting further the study of manganese-induced ferromagnetism on Ge(001), have resulted in obtaining a magnetic phase separated from vacuum by a nearly perfect Ge(001) surface. This system might be a prototype for further applications in view of the integration of magnetic systems with Si microelectronics, by taking into account that magnetic electrodes on Si are strongly reactive and therefore useless for spin injection. Note also the recent result obtained for synthesis of anisotropic magnetic layers by a similar procedure, using Fe instead of Ge [24]. Hence, the relative ease to synthesize the Ge(001) surface is a promising template for magneto-electronics and spintronics.

## Figures and Tables

**Table 1.** Relevant parameters obtained from the X-ray photoelectron spectroscopy (XPS) data analysis represented in Figure 5: energies and integral amplitudes for each separate component used in the fit. The corrected amplitudes (*A*_corr_) are obtained by dividing the integral amplitudes (*A*) by the Wagner atomic sensitivity factors [60]. The binding energies (BE) represented are that of the maximum angular moment line from each doublet: *j*_max_ = 3/2 for 2p states and 5/2 for 3d states. Experimental errors are of the order of ± the least significant digit of each binding energy or integral amplitude.

Sample	Level	Ge 3d			Ge 2p			Mn 2p		

	Component	(1)	(2)	(3)	(1)	(2)	(3)	(1)	(2)	(3)
Clean	BE *j*_max_(eV)	29.413	29.829	28.820	1217.62	1218.64	1216.37	–	–	–
	*A*(eV·kcps)	8.70	0.94	0.33	175.42	12.36	9.43	–	–	–

Ge(001) (2 × 1)	*A*_corr_(eV·kcps)	22.89	2.47	0.87	19.17	1.35	1.03	–	–	–
	*A*_corr, total_		26.23			21.55			–	

MnGe (001)	BE *j*_max_ (eV)	29.243	29.886	–	1217.40	1218.55	–	638.50	639.24	640.61
	*A*(eV·kcps)	4.33	0.49	–	110.66	7.52	–	4.29	7.64	5.87
	*A*_corr_(eV·kcps)	11.39	1.29	–	12.09	0.82	–	1.65	2.94	2.26
	*A*_corr, total_		12.68			12.91			6.85	
